# Interstitial fluid transport in linea alba is involved in acupuncture-induced attenuation of ovarian hypofunction in aged rats

**DOI:** 10.3389/fendo.2025.1579031

**Published:** 2025-05-08

**Authors:** Yaoyao Zhu, Ge Lu, Weixin Li, Chenchen Su, Pengfei Du, Shuyou Wang, Li Yang, Huanfang Xu, Yigong Fang, Xiaojing Song

**Affiliations:** Institute of Acupuncture and Moxibustion, China Academy of Chinese Medical Sciences, Beijing, China

**Keywords:** interstitial fluid, acupuncture, ovarian aging, hypothalamic-pituitary-ovarian axis, traditional Chinese medicine

## Abstract

**Background:**

Age-related ovarian dysfunction, characterized by declining follicular reserve and hormonal imbalance, poses a significant challenge in reproductive endocrinology, severely compromising fertility and quality of life. Although acupuncture at conception vessel acupoints is an effective therapeutic approach for managing ovarian dysfunction, the biological underpinnings bridging peripheral stimulation to systemic endocrine regulation remain elusive. Emerging evidence has highlighted interstitial fluid (ISF) dynamics as a potential mediator of mechanotransduction. However, it remains to be elucidated whether ISF transport in the linea alba (abdominal trajectory of conception vessel) influences ovarian function, and whether acupuncture at conception vessel acupoints exerts its therapeutic effects through ISF-mediated substance transport along the linea alba.

**Methods:**

We used fluorescence imaging to observe ISF migration patterns in the linea alba of rats, where conception vessel acupoints are located. In addition, we investigated the ovarian functional changes post-ISF obstruction and evaluated the therapeutic effects of acupuncture on ovarian dysfunction in aged rats, both in unblocked and ISF-blockade conditions.

**Results:**

Fluorescence imaging revealed ISF transport along the linea alba, with tracers migrating linearly, a pattern abolished by surgical blockade. Abnormal ISF transport in the linea alba may affect the homeostasis of reproductive hormone levels. Serum follicle-stimulating and luteinizing hormones increased while E_2_ decreased, accompanied by follicular depletion and hypothalamic-pituitary-ovarian axis dysregulation. Acupuncture at CV4 attenuated ovarian aging, elevating developmental follicle counts, reducing granulosa cell apoptosis, and restoring hypothalamic-pituitary-ovarian axis function. Crucially, these therapeutic benefits were nullified under ISF blockade conditions.

**Conclusion:**

Our results suggest that ISF transport in the linea alba may serve as an important mechanistic pathway bridging acupuncture stimulation to ovarian regulation. Our study not only provides a mechanistic basis for the clinical efficacy of acupuncture in treating ovarian disorders but also identifies ISF dynamics as a novel therapeutic target in age-related ovarian dysfunction.

## 1 Introduction

The female reproductive system is one of the first organ systems to show signs of aging. Female fertility begins to decline in the mid-thirties and ceases entirely at menopause (1). Over the past few decades, the global average age at childbearing has risen significantly, with an increasing proportion of first-time mothers aged over 35 years (2, 3). Conceiving and achieving a successful pregnancy become increasingly challenging with age, as many individuals attempt to have biological children during a period of declined fertility. The societal implications of female reproductive aging are significant, driven by delayed childbearing and the rising prevalence of age-related infertility. This trend highlights a growing societal challenge: delayed childbearing coincides with age-related infertility. Thus, preserving ovarian function and delaying reproductive aging have become critical issues in reproductive medicine.

Acupuncture, a cornerstone of traditional Chinese medicine (TCM), can enhance fertility in women with ovarian dysfunction by improving follicular development (4–9). However, the fundamental question of how peripheral mechanical stimulation at acupoints translate into systemic endocrine responses to regulate reproductive function remains unanswered. Although neuroendocrine pathways and local cytokine release have been proposed, the precise mechanisms linking acupoint stimulation to hypothalamic–pituitary–ovarian (HPO) axis modulation are poorly characterized. A critical knowledge gap persists regarding the signal propagation pathways—whether through neural reflexesor dynamic crosstalk between peripheral and central systems. Conception vessel, a key meridian governing reproductive physiology, anatomically coincides with the linea alba. Our prior research revealed the presence of fluid within the interstitial spaces of the linea alba in rats (10). These structures exhibit unique microscopic features, including abundant fibrous components arranged parallel to the long axis of the body, which facilitate the low-resistance pathways for interstitial fluid (ISF). Borroto-Escuela et al. (11) propose that volume transmission along acupuncture meridians, mediated by the diffusion and flow of interstitial fluid, may facilitate the propagation of extraneuronal signals, thereby offering a novel mechanistic framework for elucidating the functional basis of meridian systems in traditional Chinese medicine. This transmission mechanism enables bidirectional communication between meridians and supports long-range signal integration, providing a rationale for the distant regulatory effects of acupuncture. Based on these findings, we hypothesized that the role of the conception vessel in regulating reproductive function may be closely tied to the physiological properties of interstitial transport in the linea alba.

The concept of “interstitial stream” was recently introduced to describe a horizontal fluid pathway that enables connections between organs and tissues (12). Substance transport via the interstitial stream, including material transfer, energy transduction, and information transmission, may serve as a functional bridge that integrates biological systems. ISF, primarily composed of bulk water, allows the diffusion or directed flow of various cells, biological macromolecules, micromolecules, and ions (13). For example, Nedergaard et al. (14) provided direct evidence of ISF exchange between cerebrospinal fluid and brain ISF. Using two-photon imaging, they observed fluid transport behavior within the perivascular spaces of intracranial blood vessels in mice. Similarly, Dong et al. (12) described long-distance ISF transport in connective tissues of venus tunica adventitia, which is mediated by porous media at the mesoscopic scale, coining the term “interstitial stream” to characterize this phenomenon. However, whether ISF is involved in acupuncture-mediated ovarian regulation remains unexplored.

Acupuncture treatments targeting specific acupoints on the conception vessel have demonstrated efficacy in treating reproductive dysfunction (15–17). Guanyuan (CV4), the conception vessel’s cardinal reproductive acupoint located along the linea alba, is widely recognized as an effective site for treating ovarian dysfunction (18–20). Therefore, we hypothesize that acupuncture at CV4 exerts its therapeutic effects through ISF-mediated substance transport along the linea alba. This study aimed to explore the characteristics of ISF transport in the linea alba interstitium, investigate its role in maintaining ovarian homeostasis and the mechanisms by which acupuncture at CV4 alleviates ovarian aging. Understanding ISF transport may provide a biological basis for the therapeutic effects of acupuncture on ovarian function.

## 2 Results

### 2.1 Characteristics of fluorescein sodium migration after injection into the interstitial structure of the linea alba

Fluorescein sodium solution was injected into the interstitial structure of the linea alba to trace ISF behavior in live rats. Fluorescein sodium was selected for its properties as a water-soluble, nonspecific “water indicator.” It can permeate from capillaries into tissues, integrate with ISF, and accumulate in regions with more interstitial space and lower flow resistance, following the flow pattern of ISF.

Twenty minutes after fluorescein sodium injection, rats injected at the site of the linea alba (INJ group) exhibited linear migration of the tracer along the abdominal midline. However, when ISF transport in the linea alba was surgically blocked, the migration of fluorescein sodium was disrupted. In contrast, rats injected at a site 10 mm lateral to the ventral midline (INJ-control group) demonstrated a punctate, circular diffusion pattern centered at the injection site. These results indicate that ISF transport in the linea alba runs parallel to the midline and may function as a pathway for substance transport.

### 2.2 Target organs for substance transport through ISF

**Figure 1:**
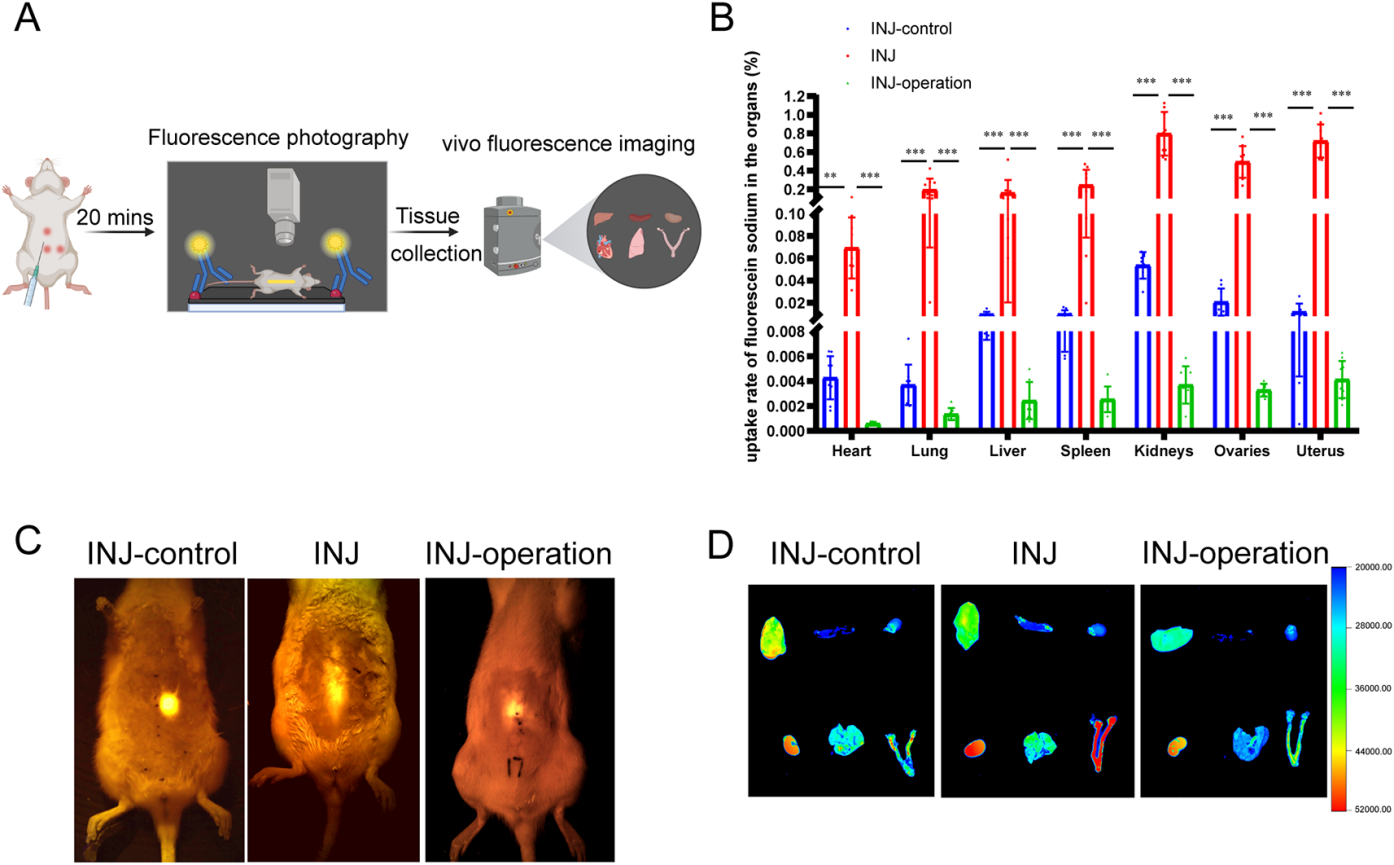
Migratory route and target organs of ISF in the linea alba. **(A)** Schematic illustration of the experimental design used to observe ISF transport behavior in the linea alba (created on https://BioRender.com). **(B, D)** Uptake of ISF in organs observed using *in vivo* fluorescence imaging (n = 9). The order of organ arrangement is: liver, spleen, heart, kidneys, lungs, uterus, and ovaries. **(C)** Tracking of fluorescein sodium injection-labeled ISF in the linea alba captured using fluorescence photography. **P < 0.01, ***P < 0.001.

To investigate whether the ISF of the linea alba is transported to internal organs, we examined the distribution of fluorescein sodium in various organs (**Figure 1A**). The kidneys, ovaries, and uterus exhibited the highest uptake of the tracer across all groups (**Figures 1B–D**). These results suggest that ISF in the linea alba can be transported to reproductive organs. Additionally, the linea alba pathway aligns with the meridian route of the conception vessel in the abdominal region, which is traditionally associated with reproductive function in TCM. Based on these observations, we hypothesized a specific correlation between ISF transport in the linea alba and reproductive function.

### 2.3 Occlusion of ISF transport compromises ovarian histology and reserve function

**Figure 2:**
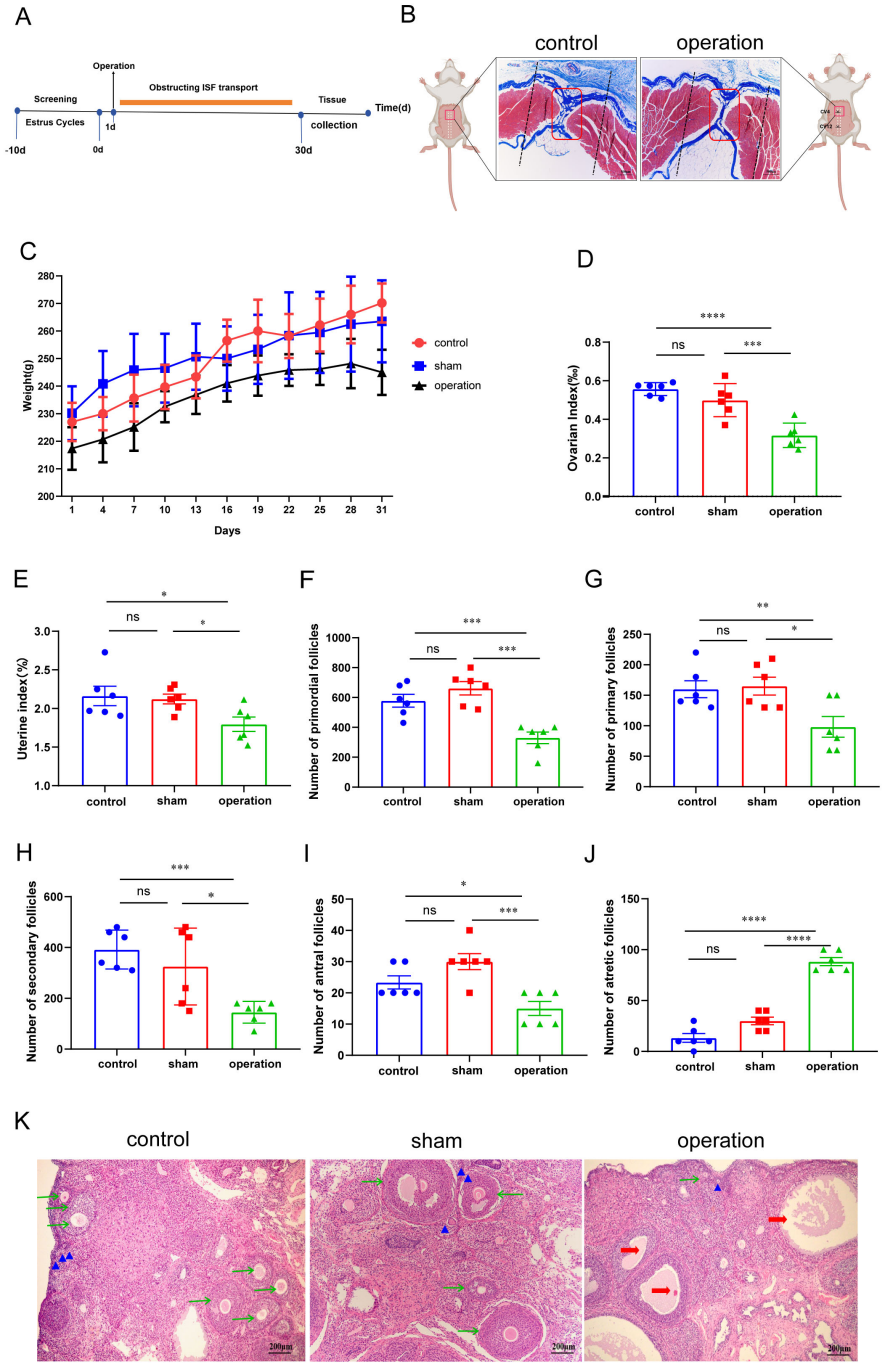
Effect of blocking ISF transport in the linea alba on ovarian histology. **(A)** Schematic illustration of the experimental design for ISF transport blockade in the linea alba. **(B)** Representative image of the interstitial structure of the linea alba. Scale bar: 500 µm. The black dotted line indicates the needle insertion and withdrawal points for the blockade operation. **(C)** Effect of ISF transport blockade in the linea alba on body weight (n = 6). **(D, E)** Ovarian and uterine indices measured (n = 6). **(F–J)** Counts and comparisons of follicles at different developmental stages (n = 6). **(K)** Histological changes in ovaries analyzed using hematoxylin and eosin (H&E) staining. Scale bar: 200 µm. Blue triangles represent primordial follicles, green arrows represent secondary follicles, and red arrows represent atretic follicles. ns: *P >* 0.05; *P < 0.05, ***P < 0.001, ****P < 0.0001.

To explore the role of ISF transport in the linea alba in ovarian function, female rats underwent surgical occlusion of ISF transport in the linea alba (**Figure 2A**). Masson’s staining revealed notable structural differences between the control and operation groups (**Figure 2B**). In the control group, longitudinally distributed fibrous connective tissue along the midline was stained blue, with relatively large interstitial spaces between the fibers. In contrast, the operation group displayed narrower interstitial spaces in the midline fibrous tissue.

Throughout the study, rats with blocked ISF transport exhibited significantly slower body weight gain compared to those in the sham group (**Figure 2C**). To directly evaluate the impact of the ISF transport blockade on ovarian function, ovarian and uterine indices were assessed, in addition hematoxylin and eosin staining was performed to analyze ovarian morphology and follicular development. Rats in the operation group exhibited lower ovarian and uterine indexes (**Figures 2D, E**, respectively) than that of the sham group. Ovarian follicles, the functional units of the ovary, consist of an oocyte surrounded by supporting granulosa and theca cells. A histological analysis revealed impaired follicular morphology and a decreased number of primordial, primary, secondary, and antral follicles in the operation group (**Figures 2F–I**). Furthermore, a notable increase in the number of atretic follicles in the operation group was observed compared to those in the sham group (**Figures 2J, K**). These results suggest that blocking ISF transport in the linea alba adversely affects ovarian histological structure and reserve function.

### 2.4 Occlusion of ISF transport diminishes ovarian endocrine function

**Figure 3:**
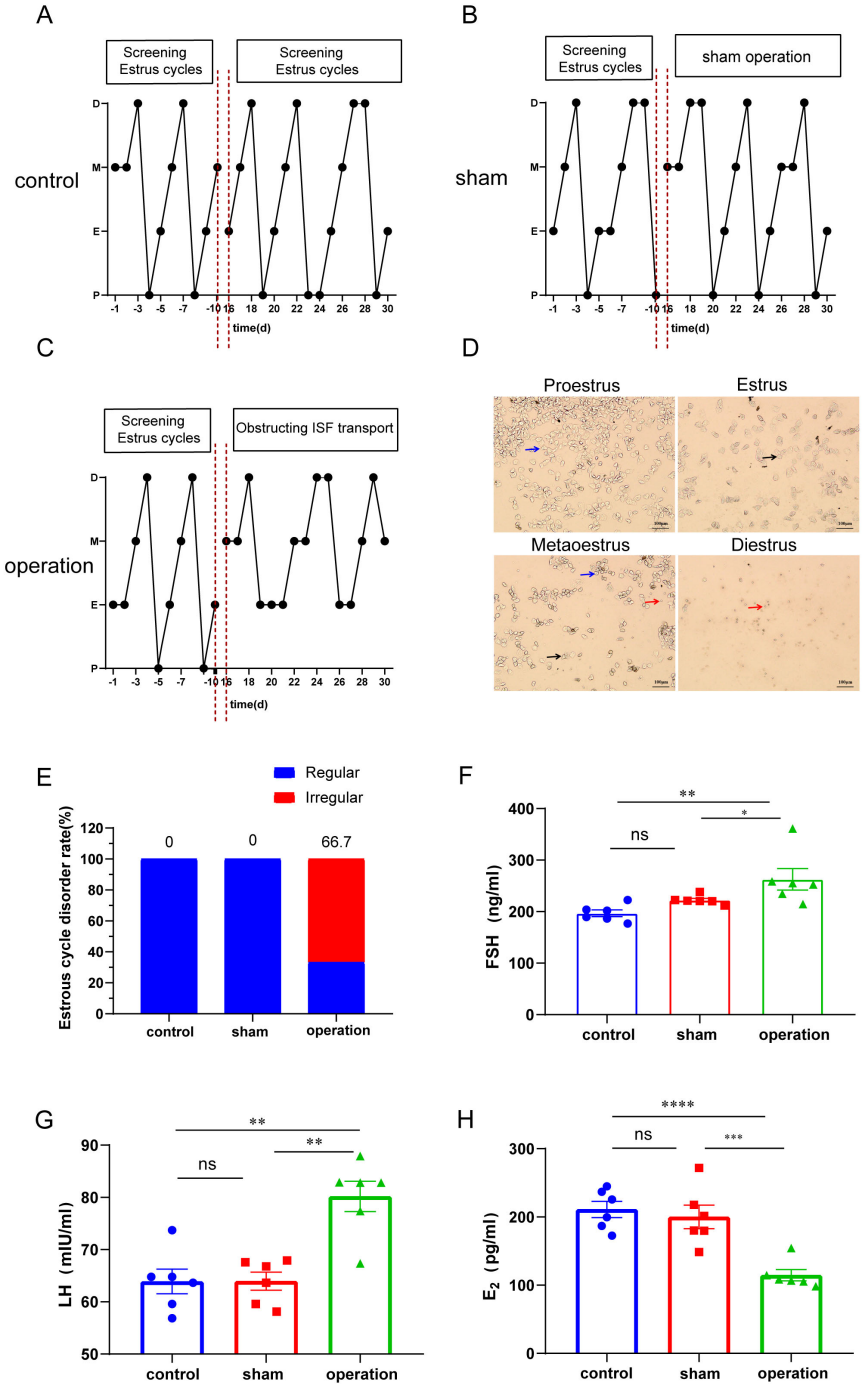
Occlusion ISF transport in the linea alba induces irregular estrous cycles and disrupts serum hormone balance. **(A–C)** Representative images of estrous cycles. **(D)** Vaginal cytology images. Blue arrows represent nucleated epithelial cells, black arrows represent flaky enucleated keratinized cells, and red arrows represent white blood cells. **(E)** Rate of estrous cycle disorders. **(F–H)** Serum levels of FSH **(F)**, luteinizing hormone (LH) **(G)**, and E_2_ **(H)** (n = 6). Scale bar: 100 µm. ns: *P >* 0.05; **P < 0.01, ****P < 0.0001.

To assess the impact of ISF transport blockade on ovarian endocrine function, we monitored the estrous cycle and hormone levels. As shown in **Figures 3A–D**, all rats had a regular estrous cycle before the intervention. After blocking the ISF transport, the operation group rats exhibited disruptions in their estrous cycle sequence and a prolonged duration in the estrous phase (**Figure 3C**).Vaginal smear analysis showed that none of the control rats exhibited irregular estrous cycles, whereas 66.7% of rats in the operation group experienced cycle irregularities (**Figure 3E**). Serum levels of estradiol (E_2_), follicle-stimulating hormone (FSH), and luteinizing hormone (LH) were measured using enzyme-linked immunosorbent assays. Compared with sham group rats, operation group rats showed a significant increase in serum FSH (**Figure 3F**) and LH (**Figure 3G**) levels, in addition to a significant decrease in serum E_2_ levels (**Figure 3H**) after 30 days of surgical ISF transport blockade. To exclude surgery-induced inflammation as a confounding factor, serum IL-6 and PGE2 levels were measured via ELISA. No significant differences were observed between the operation and control groups (**Supplementary Figure S1**), confirming minimal inflammatory impact from the surgical procedure.

### 2.5 Occlusion of ISF transport in the linea alba disrupts the HPO axis

**Figure 4:**
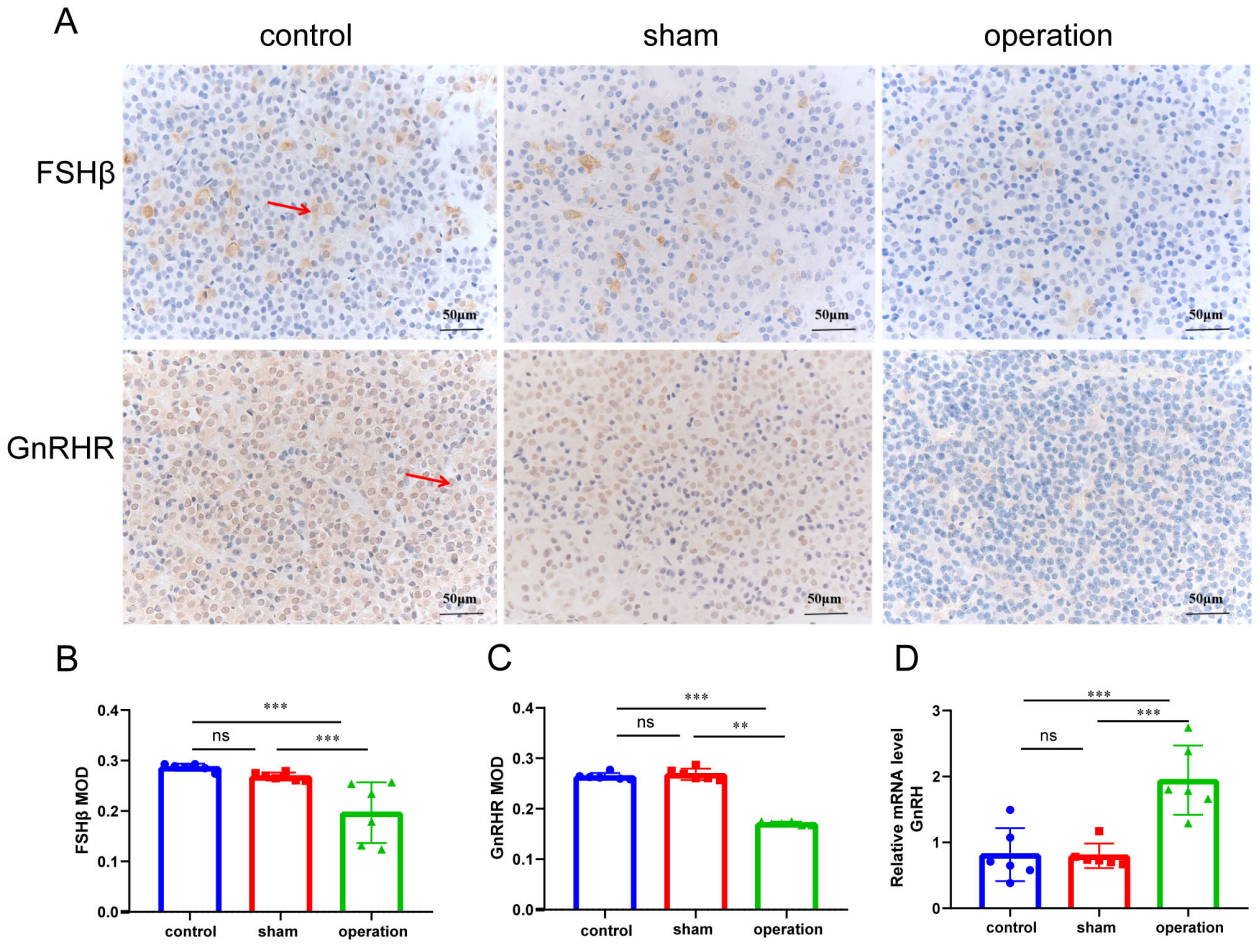
Occlusion of ISF transport in the linea alba disrupts the HPO axis. **(A)** Representative immunohistochemistry images of FSHβ and GnRHR. Scale bar: 20 µm. **(B, C)** MODs of FSHβ and GnRHR expression (n = 6). **(D)** GnRH mRNA expression in the hypothalamus (n = 6). ns: *P >* 0.05; ***P < 0.001.

The HPO axis plays a pivotal role in maintaining the balance of sex hormone levels and promoting follicular development in females. The hypothalamus secretes Gonadotropin-Releasing Hormone (GnRH) in pulses, which stimulates the pituitary gland to release FSH. FSH acts on the ovaries, promoting E_2_ secretion and follicular development. To evaluate the impact of ISF transport blockade on the HPO axis, we analyzed the levels of FSHβ, GnRHR, and GnRH. Rats in the operation group exhibited decreased levels of pituitary FSHβ and GnRHR proteins (**Figures 4A–C**), along with increased GnRH mRNA expression in the hypothalamus (**Figure 4D**). These findings suggest that the surgical occlusion of ISF transport in the linea alba may disrupt the hormonal regulation of the HPO axis, contributing to ovarian dysfunction.

### 2.6 Occlusion of ISF transport in the linea alba weakens the effectiveness of acupuncture in improving ovarian function in aged rats

**Figure 5:**
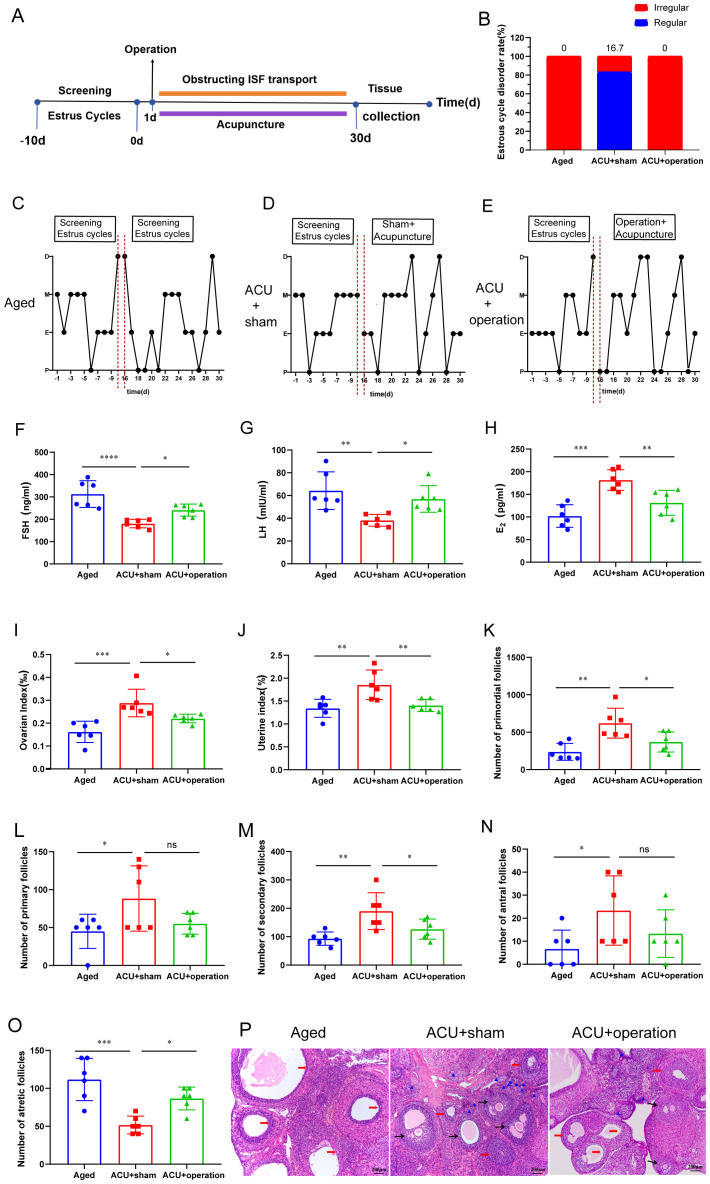
Occlusion of ISF transport inhibits the effects of acupuncture on improving ovarian function. **(A)** Schematic illustration of the experimental design used for assessing the impact of ISF transport occlusion on the therapeutic effects of acupuncture. **(B)** Rate of estrous cycle disorders (n = 6). **(C–E)** Representative images of estrous cycles. **(F–H)** Serum levels of FSH **(F)**, luteinizing hormone (LH) **(G)**, and E_2_ **(H)** (n = 6). **(I)** Ovarian index (n = 6). **(J)** Uterine index (n = 6). **(K–N)** Numbers of primordial **(K)**, primary **(L)**, secondary **(M)**, and antral follicles **(N)** (n = 6). **(O)** Number of atretic follicles (n = 6). **(P)** Representative histological structure of ovaries. Scale bar: 200 μm. ns: *P >* 0.05; *P < 0.05, **P < 0.01, ***P < 0.001.

**Figure 6:**
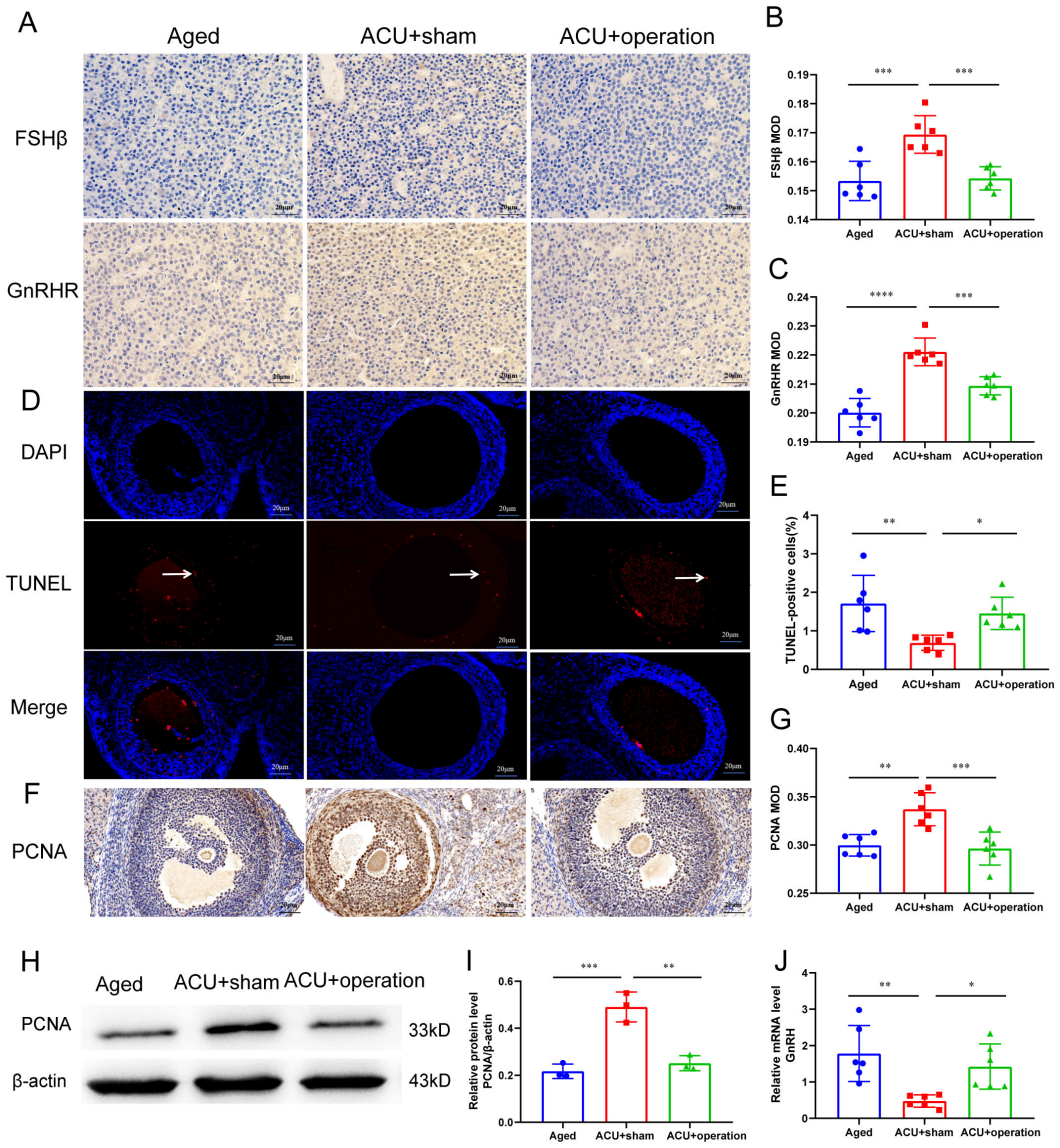
Occlusion of ISF transport reverses the benefits of acupuncture on granulosa cell development and HPO axis stability. **(A–C)** Quantitative analysis of FSHβ and GnRHR expression using immunohistochemistry (IHC), in addition to IHC staining images of FSHβ and GnRHR in ovarian tissues. Scale bar: 20 µm (n = 6). **(D)** Representative TUNEL staining images of ovarian tissues. Scale bar: 20 µm (n = 6). White arrows represent TUNEL-positive cells. **(E)** Percentage of TUNEL-positive cells (n = 6). **(F, G)** IHC analysis of PCNA expression levels and representative IHC staining images in ovarian tissue. Scale bar: 20 µm (n = 6). **(H, I)** Western blot analysis of ovarian PCNA levels and representative Western blot images of PCNA expression (n = 3). **(J)** Quantitative reverse transcription polymerase chain reaction analysis of pituitary GnRH expression (n = 6). *P < 0.05, **P < 0.01, ***P < 0.001.

Acupuncture is widely recognized as a therapeutic approach to enhance ovarian function in TCM. To investigate its effects on ovarian function in 9-month-old rats, their estrous cycles were first analyzed (**Figure 5A**). Acupuncture significantly reduced the incidence of estrous cycle disorder (**Figure 5B**). Compared with aged and ACU+operation groups, 9-month-old ACU+sham-treated rats exhibited a notable shift (**Figures 5C–E**). Specifically, the cycles transitioned from a state of sequential disorder or prolonged persistence in the estrous period to a sequential appearance in each stage of the estrous cycle. Serum hormone analysis displayed that, compared to the aged group, the ACU+ Sham group exhibited elevated levels of E_2_ and LH, in addition to a notable reduction in FSH levels (**Figures 5F–H**). Furthermore, ovarian and uterine indices were significantly higher in the ACU+sham group than in the aged group (**Figures 5I, J**). Histological analysis revealed that acupuncture significantly increased the number of normal follicles (**Figures 5K–N**) and reduced the number of atretic follicles (**Figures 5O, P**). In addition, acupuncture attenuated the disturbance in the HPO axis (**Figures 6A–C, J**) and apoptosis of ovarian granulosa cells (**Figures 6D, E**), and increased PCNA protein expression, a marker of cell proliferation, in 9-month-old rats (**Figures 6F–I**). Collectively, these results suggest that acupuncture improves ovarian function in 9-month-old rats.

To further investigate the underlying mechanisms, the role of ISF transport in the linea alba in mediating the protective effects of acupuncture was examined. These results suggest a specific relationship between ISF transport in the linea alba and ovarian function. Accordingly, we investigated whether ISF transport plays a role in the protective effects of acupuncture on ovarian function. To assess the impact of ISF transport occlusion on the therapeutic benefits of acupuncture, rats subjected to ISF transport occlusion underwent a 30-day acupuncture intervention. Compared with that of the ACU+sham group, the ACU+operation group showed an increased incidence of estrous cycle disorders (**Figure 5B**). Serum hormone analysis revealed elevated FSH and LH levels (**Figures 5F, G**) and reduced E_2_ levels (**Figure 5H**) in the ACU+operation group. Subsequently, the effects of ISF transport occlusion on ovarian histological restoration was investigated in 9-month-old rats. The ACU+operation group exhibited significantly lower ovarian and uterine indices than the ACU+sham group (**Figures 5I, J**). Histological analysis revealed that ISF transport occlusion reduced the number of primordial, primary, secondary, and antral follicles (**Figures 5K–N**) while increasing the number of atretic follicles (**Figure 5O**). In addition, the impact of ISF transport occlusion on the HPO axis was assessed by analyzing the FSHβ, GnRHR, and GnRH levels. Compared to those of the ACU+sham group, the FSHβ and GnRHR levels in the hypothalamus were lower in the ACU+operation group, whereas the pituitary GnRH level was higher (**Figures 6A–C, J**).

To further support these findings, granulosa cell apoptosis in ovarian tissues was analyzed using the Terminal dUTP Nick End Labeling(TUNEL) assay. The ACU+operation group showed a higher number of TUNEL-positive cells than the ACU+sham group, both quantitatively and qualitatively (**Figures 6D, E**). Additionally, protein expression of PCNA, a marker of cell proliferation, in the ACU+operation group was significantly lower than that in the ACU+sham group (**Figures 6F–I**). These findings indicate that ISF transport in the linea alba may play a crucial role mediating the therapeutic benefits of acupuncture on ovarian function.

## 3 Discussion

To our knowledge, this study provides the first mechanistic evidence of the involvement of ISF transport in the linea alba in the therapeutic effects of acupuncture at CV4 on ovarian function in aged rats. The directional migration of fluorescein sodium along the linea alba, coupled with its obstruction-induced abolition, demonstrates that this anatomically specialized structure serves as a conduit for mechanotransduction. Importantly, surgical blockade of ISF transport not only precipitated ovarian dysfunction, evidenced by follicular depletion, hormonal imbalance, and HPO axis disruption, but also nullified acupuncture’s restorative effects. Thus, ISF transport may act as an important component of CV4’s therapeutic mechanism. These findings address a critical gap in understanding regarding how peripheral acupoint stimulation translates into ovarian endocrine regulation.

Smith et al. (21) were the first to identify conduction channels between interstitial structures and tissues. Termed “tissue channels,” these conduits possess a randomly distributed, porous, grid-like structure that facilitates the flow of materials, energy, and information in fluid-rich tissues. Similarly, Li et al. (22) used radionuclides to trace migration pathways along meridians, finding a close relationship with the vascular system, but not with nerves. Zhang et al. (23) further identified low hydraulic resistance channels associated with ISF in miniature pigs, though the specific anatomical structure and underlying mechanisms remain unclear. Yao et al. (24) simulated directional interstitial flow and proposed that ISF may create a mechanical environment that guides cellular activities. Moreover, the role of mast cells in qi generation suggests a close connection between ISF and TCM principles. These findings collectively highlight the involvement of interstitial channels and ISF transport in various physiological processes.

Dong et al. (25) demonstrated long-range fluid transport behavior in the connective tissues of the tunica adventitia of veins, coining the term “interstitial stream” to describe this efficient long-distance transport pathway. Our research group previously reported the presence of a specialized interstitial channel in the ventral midline which facilitates substance transport (26). In the present study, we observed similar long-range fluid transport behavior within the interstitial structure of the linea alba. Morphological observations showed that the interstitial tissue of the linea alba contains larger interstitial spaces than those of the surrounding muscle tissue. Subsequently, we employed visual methods to investigate the effects of blocking ISF transport in the linea alba of living rats. In accordance with a previous study (27), fluorescence imaging confirmed the linear migration of the tracer along the midline; however, this migration was blocked upstream by ISF transport obstruction, consistent with fluid transport behavior in the interstitial structure.

The transport behavior of ISF in interstitial structures is generally classified into three forms: material transfer, energy transduction, and information transmission, collectively referred as “substance transport.” Growing attention has been directed toward understanding and regulating ISF transport, particularly in the diagnosis and treatment of complex diseases (28, 29). The conception vessel plays a central role in regulating reproductive functions which is a fundamental concept in TCM. Previous studies, including ours, have demonstrated that acupuncture at conception vessel acupoints can regulate the HPO axis, modulate sex hormone secretion, mitigate ovarian dysfunction, and ultimately improve fertility outcomes (30–33). Interestingly, the abdominal course of the conception vessel coincides with the pathway of the interstitial structure in the linea alba. This intriguing overlap led us to hypothesize that the regulation of ovarian function by conception vessels may involve ISF transport in the linea alba. However, whether ISF transport in the linea alba participates in ovarian function is unclear.

In light of this, to explore the biological role of ISF in ovarian function, we conducted surgical procedures to obstruct ISF transport in the linea alba of female Sprague–Dawley(SD) rats. Compared with those of the sham group rats, the body weight of rats in the operation group increased slower, and the ovarian and uterine indices of the rats in the operation group were significantly lower. Hormonal imbalance, a hallmark of diminished ovarian function, was also observed. Specifically, ISF transport occlusion led to irregularities in the estrous cycle and serum hormone levels, a reduced number of normal follicles, and an increased number of atretic follicles. Notably, the sham group did not exhibit ovarian dysfunction induced by blocking ISF transport in the linea alba. Furthermore, there was no statistically significant differences in the levels of serum inflammatory cytokines, which are released when tissues are subjected to injury, infection, or other stimuli; therefore, we excluded postoperative inflammation as a confounding factor. These results indicate that the ovarian dysfunction was associated with obstruction of ISF transport in the linea alba.

The HPO axis is crucial for regulating reproductive function (34–36). Hypothalamic GnRH serves as the initiating signal in this axis and is secreted in a pulsatile manner into the hypothalamic-pituitary portal system. The GnRH binds to its receptor (GnRHR) on the surface of pituitary gonadotrophs, triggering the synthesis and secretion of gonadotropins, including FSH. FSH consists of an α-subunit and a β-subunit (FSHβ). Pituitary FSHβ, in particular, plays a vital role in follicular development and oocyte maturation within the ovary. Specifically, it stimulates ovarian follicle growth and granulosa cell differentiation, both of which are critical for E_2_ production and oocyte development. The intricate interplay of pituitary FSHβ and GnRHR and hypothalamic GnRH ensures the coordinated regulation of female reproductive processes. In our study, rats in the operation group exhibited reduced FSHβ and GnRHR protein levels in the pituitary gland, along with increased GnRH mRNA expression in the hypothalamus. These findings indicate that impaired ISF transport disrupts the HPO axis, thereby contributing to ovarian dysfunction. Altogether, and consistent with our hypothesis, these results confirm that ISF transport in the linea alba may be essential for maintaining ovarian function. Unfortunately, the specific mechanisms by which ISF transport in the linea alba regulates ovarian function remains unclear. The ISF in the linea alba may serve as a critical horizontal fluid connection pathway; however, research on its regulatory role in ovarian function is still in its infancy. We anticipate that future studies will provide additional insights into this topic.

Acupuncture, a key therapeutic modality in TCM, exerts protective effects on ovarian function (37–39). Previous studies have identified several potential mechanisms underlying these beneficial effects, including promoting follicle development (37), inhibiting apoptosis of ovarian granulosa cells (38), modulating mitochondrial apoptosis and autophagy (31), and influencing neuroendocrine system in the ovaries (39, 40). However, a gap remains in our understanding regarding how peripheral acupuncture stimulation leads to molecular changes within the ovaries. To address this gap, we aimed to investigate whether peripheral acupuncture stimulation at the ventral midline promotes ISF transport in the linea alba, thereby alleviating follicular development abnormalities and enhancing ovarian function. In this study, the CV4 acupoint was selected for the acupuncture intervention. The CV4, an acupoint on the conception vessel, converges with the three yin meridians of the foot and is traditionally used to regulate qi and blood within the thoroughfare and conception vessels. Bibliometric analyses have identified CV4 as the most frequently used acupoint on the conception vessel for the clinical treatment of ovarian diseases (41). Acupuncture at CV4 has been shown to regulate the HPO axis, modulate sex hormone secretion (42, 43), and mitigate ovarian dysfunction. Therefore, CV4 was selected to explore the therapeutic effects of acupuncture on ovarian function. Our data displayed that acupuncture at CV4 effectively restored ovarian hypofunction and alleviated folliculogenic disorders in aged rats. This was evidenced by increased cell proliferation markers, reduced ovarian granulosa cell apoptosis, and regulation of the HPO axis, consistent with previous findings (44, 45). Furthermore, to examine the role of ISF transport in the protective effects of acupuncture, ISF transport was surgically blocked at the linea alba site. Compared with those of the ACU+sham group rats, the number of normal follicles in the ACU+operation group was lower, the number of atretic follicles was higher, the serum E_2_ levels were lower, and the levels of FSH and LH were higher. In addition, pituitary FSHβ and GnRHR expressions were elevated, whereas hypothalamic GnRH expression was reduced. Ovarian granulosa cell apoptosis increased, and PCNA expression in the ovarian tissues was significantly reduced in the ACU+operation group. These results demonstrate that the disruption of ISF transport may weaken the beneficial effects of acupuncture on ovarian function in aged rats.

**Figure 7:**
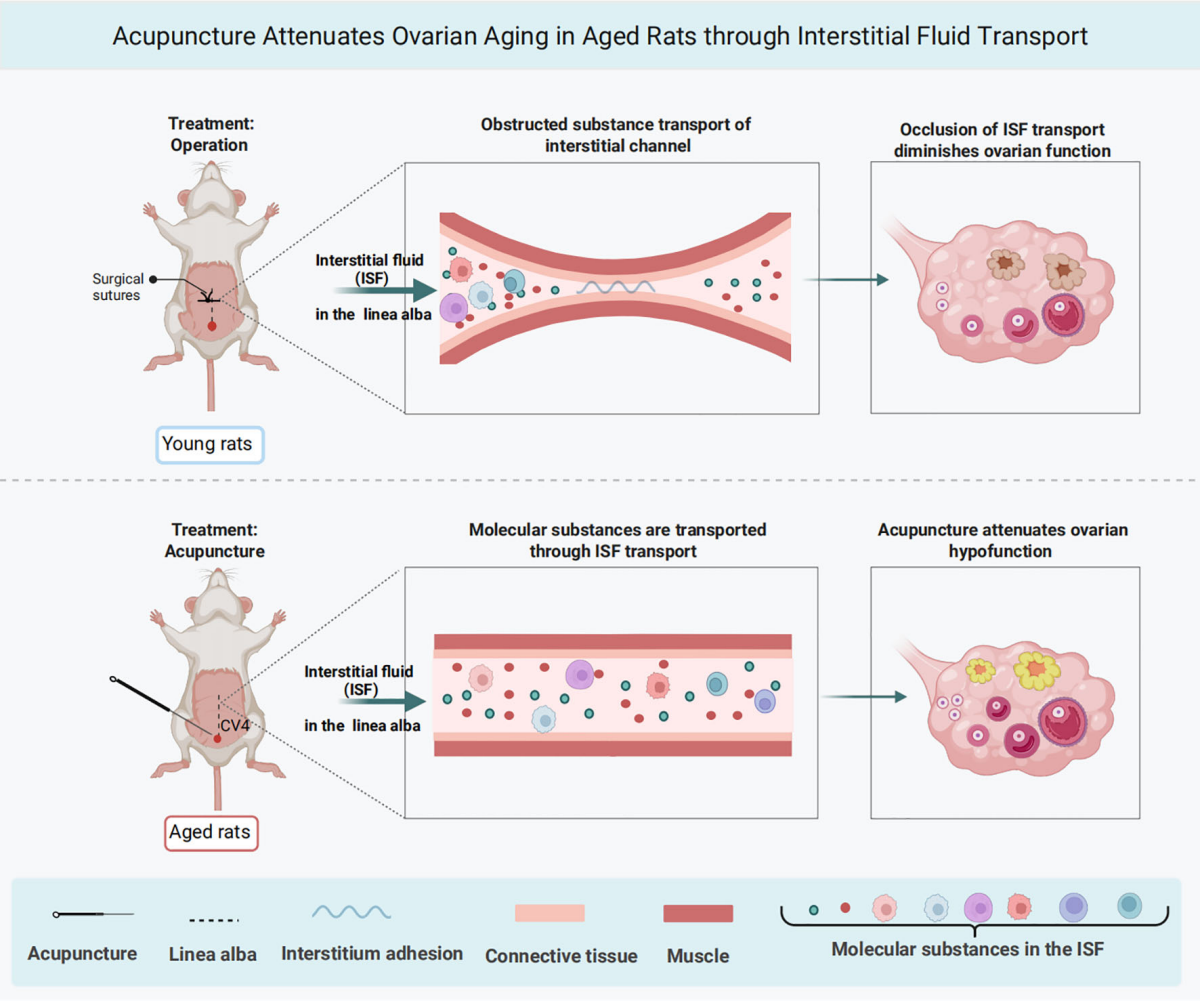
Graphical abstract. ISF transport among linea alba is involved in the regulation of ovarian function in acupuncture. Acupuncture at CV4 attenuates ovarian hypofunction in 9-month-old rats through ISF transport(created on https://BioRender.com).

Acupuncture is a physical and mechanical stimulus that induces the local production and secretion of various biochemical signals, including neurotransmitters, extraneuronal signals, the gut microbiota, and mast cells (46–50). These mechanical signals are integrated and transmitted through complex network pathways, ultimately delivering effector information to target organs and eliciting a range of biological effects. Fuxe et al. (51) proposed that volume transmission, a key communication mechanism in the central nervous system, may mediate the transport and diffusion of neurotransmitters and extraneuronal signals along extracellular (interstitial) fluid pathways, including acupuncture meridians. Both short- and long-distance acupuncture-induced volume transmission may occur along meridian channels through ISF diffusion and flow. Based on these findings, we postulate that acupuncture generates signaling substances that are transported through ISF along the linea alba, ultimately enhancing ovarian function(**Figure 7**). Impaired ISF transport within the linea alba disrupts the conveyance of these acupuncture-induced signaling substances, thereby diminishing the efficacy of acupuncture in improving ovarian function.

Although the findings of our study are quite novel, the study itself had some limitations that should be considered. This experimental was designed to demonstrate whether ISF transport in the linea alba is involved in the biological process of ovarian function regulation by acupuncture. However, many aspects remain to be elucidated, for instance, the active substances presents in the ISF of the linea alba involved in this regulatory process and their mechanism of action, differences between physiological and pathological states, whether acupuncture at the conception vessel points affects ISF transport. In future studies, we intend to conduct a more systematic research to analyze the composition of the ISF in the linea alba under physiological and pathological ovarian conditions, and after acupuncture stimulation through multi-omics techniques to elucidate the biological role of ISF transport in the maintaining ovarian function. Control experiments assessing local interstitial collagen fiber lysis need to be conducted to further verify the involvement of ISF transmission in acupuncture regulation of ovarian function.

## 4 Materials and methods

### 4.1 Animals and ethics

Female SD rats, aged 2 months (weighing 210–240 g) and 9 months (weighing 380–400 g), were provided by Beijing Vital River Laboratory Animal Technology Co., Ltd. All animals were housed in a temperature- and humidity-controlled environment with a fixed 12-h light/dark cycle. They had *ad libitum* access to standard laboratory rat pellet food and water at the Laboratory Animal Center of the Institute of Acupuncture and Moxibustion, China Academy of Chinese Medical Sciences. All animal experimental procedures were approved by the Ethics Committee of the Institute of Acupuncture and Moxibustion, China Academy of Chinese Medical Sciences (March 21st, 2024; Approval No. D2024-03-26-01).

### 4.2 Experimental design

Three separate experiments were conducted in this study.

#### 4.2.1 Experiment 1

The objective was to characterize ISF transport behavior along the linea alba and investigate whether organ targeting occurs. A total of 27 female SD rats aged 2 months with regular estrous cycles were randomly divided into three groups (n = 9 per group): injection lateral to the linea alba (INJ-control), injection at the site of the linea alba (INJ), and injection at the linea alba after surgical obstruction of ISF transport (INJ-operation). After anesthesia, the abdominal fur was shaved, and the skin was disinfected with 75% alcohol. The ventral midline, extending from the xiphoid process to the external genitalia, was divided into five equal segments. The injection site for the INJ group was designated as Point 1, located one-fifth of the distance from the xiphoid process. The INJ-control group received injections 10 mm lateral to Point 1. In the INJ-operation group, injections were performed at Pont 1; however, surgery was conducted at Point 2 (5 mm below Point 1) to block ISF transport in the linea alba. In all groups, the corresponding skin locations on the rats were punctured using a 1 mL syringe at a 45° angle to a depth of 2–3 mm. Subsequently, 0.5 mL of a 5% fluorescein sodium was slowly injected. After 20 min, fluorography was performed to trace the migration of fluorescein sodium on the body surface of the rat. Fluorescent uptake in various organs was analyzed using Carestream *In-Vivo* FX PRO Acquire software (licensed under CC-BY-NC-ND 4.0).

#### 4.2.2 Experiment 2

Experiment 1 confirmed that the ISF transport in the linea alba is characterized by targeted ovarian aggregation. Based on these findings, Experiment 2 assessed the effect on ovarian function by blocking ISF transport in the linea alba. The goal was to determine whether this directional ISF transport in the linea alba is causally associated with ovarian function maintenance. A total of 18 female SD rats aged 2 months with regular estrous cycles were randomly assigned to three groups (n = 6 per group): control group (no surgery was performed), sham group (surgery was performed 10 mm lateral to Point 2), and operation group (surgery was performed at Point 2 within the linea alba), as described in Experiment 1. In the operation group, ISF transport was blocked by surgically excising a 5 mm-wide and 5 mm-deep section of abdominal wall tissue, including the peritoneum, fibrous connective tissue, and muscle fibers, at Point 2. For the sham group, a similar surgical procedure was conducted 10 mm lateral to Point 2. The surgical blockade of interstitial transport was maintained for 30 days. On Day 31, several parameters were assessed in all groups, including the estrous cycle, serum hormone levels, ovarian index, ovarian morphology, and follicle count.

#### 4.2.3 Experiment 3

As Experiment 2 demonstrated that occlusion of ISF transport in the linea alba negatively effects on the ovarian function, Experiment 3 aimed to explore the effect of blocking ISF transport on the regulation of ovarian function by acupuncture in 9-month-old rats to understand whether substance transport in the linea alba is involved in this process at conception vessel points. Our previous study (52) and other literature (53, 54) demonstrated significant age-related ovarian function differences in female SD rats. In this experiment, 9-month-old rats were selected as experimental subjects, as it remains within the reproductive period and reflect ovarian functional decline. In total, 18 female SD rats aged 9 months were divided into three groups (n = 6 per group): Aged group (no acupuncture or surgery was performed), ACU+sham group (acupuncture treatment combined with sham surgery, 10 mm lateral to Point 2), and ACU+operation group (acupuncture combined with surgery to block ISF transport at Point 2). In both the ACU+sham and ACU+operation groups, a 20-min acupuncture treatment was administered daily at CV4 for 30 consecutive days. In the ACU+sham and ACU+operation groups, blockade surgery was performed 10 mm lateral to Point 2 and at Point 2, as in Experiment 1, respectively. The rats in the aged group were handled similarly to those in the other groups but did not receive acupuncture treatment.

### 4.3 Blocking ISF transport in the linea alba

Following isoflurane anesthesia, the abdominal skin was depilated and disinfected. Surgical sites were defined at Point 2 and 10 mm laterally to Point 2. At the site of Point 2, an area of approximately 2.5 mm × 2.5 mm was designated for needle insertion (left horizontal position) and exit (right horizontal position). The suture needle was inserted through the skin to the superior aspect of the peritoneum, thereby passing through and securing the skin, subcutaneous connective tissue, and abdominal wall muscles. The skin, subcutaneous connective tissue, and abdominal muscles were all sutured together, and the linea alba was in the center of the suture area. When ligating the sutures, the force was moderate to avoid tissue damage. Post-operation disinfection was performed using 75% ethanol. The lateral surgical site (10 mm laterally to Point 2) underwent identical procedural steps.

### 4.4 Tracing with fluorescein sodium

After anesthetizing the rats with isoflurane, they were placed in a supine position on an operating table. Fluorescein sodium (Guangxi Wuzhou Pharmaceutical Co., Ltd., Wuzhou, China) was prepared as a 1% solution in physiological saline. Using an insulin syringe, 0.1 mL/kg of this solution was injected subcutaneously at a depth of approximately 3 mm, perpendicular to the skin. Twenty minutes after the injection, *in vivo* fluorescence imaging was performed to observe the migration of fluorescein sodium on the body surface of the rat. Imaging was conducted using a Canon 5D2 camera equipped with an optical filter (570 nm), a laser sensor (0.5 mW power), and an excitation wavelength of 455 nm.

### 4.5 Distribution of fluorescein sodium in organs

Following fluorescence imaging of the tracer migration track, the rats were euthanized via the abdominal aorta. Organs, including the heart, lungs, liver lobes, spleen, kidneys, uterus, and ovaries, were promptly excised and placed in the dark chamber of a Carestream *In-Vivo* Imaging System FX PRO for fluorescence imaging. The imaging parameters included an excitation wavelength of 470 nm, an emission wavelength of 535 nm, and an exposure time of 10 s. To analyze fluorescence intensity, Carestream *In-Vivo* FX PRO Acquire software (licensed under CC-BY-NC-ND 4.0) was used. Furthermore, each organ was processed into a supernatant to isolate fluorescein sodium. Fluorescence content in the supernatants was measured using a Thermo VARIOSKAN microplate reader and SkanIt software version 2.4.3. The uptake rate of fluorescein sodium in each organ was calculated using the following equation:


$$Uptake\;rate\;=\frac{\left[\left(\frac{C_{organ}}{C_{injection}}\right)\times \;100\%\right]}{W_{organ}}$$

where C_organ_ represents the fluorescein sodium content in the organ, C_injection_ represents the total fluorescein sodium injected, and W_organ_ represents the weight of the organ.

### 4.6 Masson staining

The abdominal wall tissue centered at the surgical operation site along the ventral midline was excised with approximate dimensions of 10 mm in length, 10 mm in width, and 0.5 cm in thickness. The tissue was embedded in paraffin blocks, and 5-µm-thick sections were prepared for staining. Masson staining was performed to evaluate the morphological and structural characteristics of the interstitial tissue in the linea alba.

### 4.7 Estrous cycle

Vaginal smears were performed daily at 10:00 a.m. to monitor the estrous cycle. A pipettor was used to aspirate 200 µL of normal saline, which was then carefully injected into the vaginal canal. Vaginal secretions were collected via reaspiration and evenly spread onto glass slides for examination. The morphology of exfoliated vaginal cells was observed using a 10× magnification microscope (CKX53SF; Olympus, Tokyo, Japan) to determine the estrous cycle stage. The estrous cycle consists of four stages: proestrus, estrus, metestrus, and diestrus (55). Estrous cycle irregularities were defined as a prolonged cycle (≥6 days) or persistence in a single stage for ≥ 3 days, which are indicative of decreased reproductive function, as previously described (56).

### 4.8 Ovarian morphology and follicle counting

Ovarian tissues were fixed in 4% paraformaldehyde for 48 h, embedded in paraffin, and sectioned into 5 μm-thick slices. One section of each ovary was stained with hematoxylin and eosin to assess ovarian histomorphology and analyzed under a light microscope(Olympus BX51). Follicular classification and counting followed established criteria (57, 58), categorizing follicles into five distinct stages based on morphological and structural features. Primordial follicles were defined as oocytes enveloped by a single layer of flattened granulosa cells. Primary follicles exhibited one to two layers of cuboidal granulosa cells surrounding the oocyte. Secondary follicles displayed more than two layers of cuboidal granulosa cells without visible fluid-filled cavities. Antral follicles were characterized by a prominent cumulus oophorus complex and multiple layers of cuboidal granulosa cells. Atretic follicles were identified by degenerative features, including nuclear fragmentation of the oocyte, cytoplasmic dissolution, and a reduced granulosa cell layer. The number of antral follicles per section was recorded, and the total follicle counts was calculated by multiplying this value by five. Six samples per group were analyzed.

### 4.9 Hormone Assays

Rats in the diestrus stage were anesthetized with 2% pentobarbital sodium (0.25 mL/100 g) and subjected to abdominal aortic blood collection. Serum was obtained through centrifugation at 3,500 × *g* for 15 min at 4°C, and the supernatant was stored for subsequent hormone analysis. Serum concentrations of E_2_, FSH, and LH were quantified using enzyme-linked immunosorbent assay (ELISA) kits(E_2_: SEKR-0107; FSH: SEKR-0090; LH: SEKR-0091; Solarbio, Beijing, China), according to the manufacturer’s protocols. The assay included standard, blank, and sample wells (all in duplicate). After sealing, the microplate was incubated at 37°C. Subsequent steps involved sequential addition of biotinylated antibody working solution, enzyme conjugate working solution, and substrate solution. The reaction was terminated using stop solution, and the optical density (OD) values was immediately measured at 450 nm on a microplate reader. Data were corrected by subtracting the blank well OD values, and standard curves were generated to calculate hormone concentrations in the samples.

### 4.10 TUNEL staining

TUNEL staining was performed to detect apoptotic ovarian cells using the TMR (red) TUNEL Cell Apoptosis Detection Kit (G1502, Servicebio, Wuhan, China). Nuclei were counterstained with DAPI (1 µg/mL). For each sample, six random sections per slice were analyzed at 400 × magnification, and the apoptotic index (percentage of TUNEL-positive cells) was calculated using ImageJ software.

### 4.11 Immunohistochemistry staining

Tissue sections underwent standard preparation steps, including dehydration, antigen retrieval, endogenous peroxidase inhibition, and serum blocking. Paraffin-embedded sections were incubated overnight at 4°C using the following primary antibodies: anti-FSHβ (1:200, ab281562, Abcam, Shanghai, China), anti-GnRHR (1:400, ab202848, Abcam), and anti-PCNA (1:600, ab92552, Abcam). After incubation, sections were treated with the corresponding secondary antibodies (1:500, ab6721; Abcam) for 1 h at 22°C, followed by diaminobenzidine staining and hematoxylin counterstaining. Digital scanning of the slides was performed using a Pannoramic 250 FLASH scanner (Pannoramic MIDI, 3DHISTECH, Budapest, Hungary).

### 4.12 Western blotting

Ovarian tissues were lysed in RIPA buffer containing a protease inhibitor cocktail (BL504A, Biosharp, Hefei, China). The samples were homogenized using a tissue grinder, lysed on ice for 30 min, and then centrifuged at 12,000 rpm for 20 min at 4°C. Protein concentrations were measured, and equal amounts of protein (20 μg per sample) were separated using 10% SDS-PAGE and transferred onto polyvinylidene fluoride membranes (ISEQ00010, Millipore, Burlington, MA, USA). Membranes were incubated overnight at 4°C with the following primary antibodies: anti-PCNA (1:2,000, ab92552, Abcam). After washing, the membranes were incubated with secondary goat anti-rabbit IgG-HRP (1:10,000, 111-035-003, Jackson ImmunoResearch Laboratories, West Grove, PA, USA) for 1 h at 22°C. Signals were visualized using an enhanced chemiluminescence kit (WBKLS0500, Millipore). Three samples from each group were analyzed. The protein levels were normalized to those of β-actin (1:10,000, 66009-1-Ig, Proteintech, Wuhan, China). Densitometric analysis was conducted using ImageJ software.

### 4.13 Quantitative reverse transcription polymerase chain reaction

Total RNA was extracted from ovarian tissue using RNA extraction buffer (Servicebio). Reverse transcription of RNA into cDNA was performed in a 20 μL reaction volume using the SweScript All-in-One RT SuperMix for qPCR (G3337, Servicebio). Relative quantification of mRNA expression was performed using 2×Universal Blue SYBR Green qPCR Master Mix (G3326; Servicebio) and a quantitative PCR instrument. Primer sequences for the PCR reactions are listed in **Table 1**. The housekeeping gene *Gapdh* was used as the internal control. Relative mRNA expression levels were calculated using the 2^−ΔΔCT^ method.

**Table 1:** Primer sequences used for quantitative reverse transcription polymerase chain reaction.

Gene symbol	Forward primer	Reverse primer	Product length (bp)
*GnRH*	TGGTATCCCTTTGGCTTTCACA	GTCAACAGAACAACAGCGGC	155
*Gapdh*	CCATGGAGAAGGCTGGGG	CAAAGTTGTCATGGATGACC	138

### 4.14 Statistical analysis

Statistical analyses were performed using SPSS statistical software (version 26.0; IBM, Armonk, NY, USA). Data are expressed as the mean ± standard deviation. Two-tailed Student’s t-test was used for comparisons between two groups, while one-way analysis of variance was used for comparisons among multiple groups. Count data are expressed as percentages and were analyzed using Fisher’s exact test. A P < 0.05 was considered statistically significant.

## 5 Conclusions

Our study provides the first evidence that interstitial transport along the linea alba may mediate the therapeutic effects of acupuncture at CV4 on ovarian function in aged rats. The blockade of ISF transport may diminish ovarian function, underscoring the importance of fluid movement in the interstitial space. Although the precise signaling mechanisms remain to be fully elucidated, our findings highlight the critical role of ISF transport in facilitating acupuncture-induced biological effects. Future research should focus on characterizing at the molecular level signaling molecules within the ISF that mediate these effects, coupled with biophysical modeling to quantify ISF flow dynamics in meridian pathways.

## Data Availability

All relevant data is contained within the article: The original contributions presented in the study are included in the article/supplementary material, further inquiries can be directed to the corresponding authors.
