# Supplementary material for: Interstitial fluid transport in linea alba is involved in acupuncture-induced attenuation of ovarian hypofunction in aged rats
Source: Front Endocrinol (Lausanne). 2025 May 8;16:1579031. doi: 10.3389/fendo.2025.1579031 (PMC12094915; doi:10.3389/fendo.2025.1579031)
Supplement: Supplementary file 1 [file DataSheet1.pdf]

Supplementary Material

1 Supplementary Figures

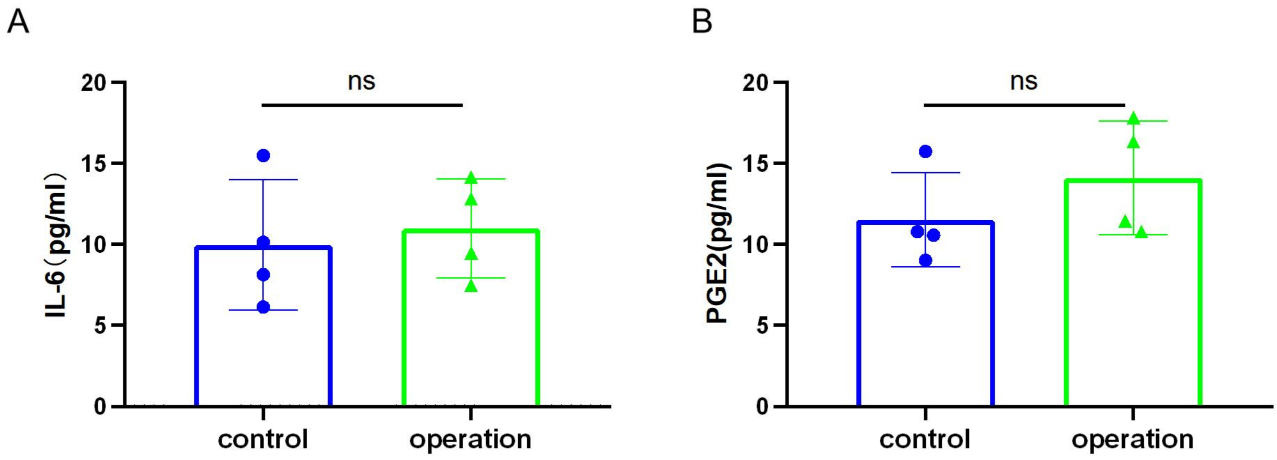

Supplementary Figure 1. Occlusion ISF transport in the linea alba does not induce detectable inflammatory responses. Serum levels of IL-6 (A) and PGE2 (B), n=4, ns: $P > 0.05$.
